# Machine learning methods to predict particulate matter PM _2.5_

**DOI:** 10.12688/f1000research.73166.1

**Published:** 2022-04-11

**Authors:** Naveen Palanichamy, Su-Cheng Haw, Subramanian S, Rishanti Murugan, Kuhaneswaran Govindasamy

**Affiliations:** 1 Faculty of Computing and Informatics, Multimedia University, Cyberjaya, Selangor, 63100, Malaysia; 2 Department of Electrical Engineering, Annamalai University, India, Chidambaram, Tamil Nadu, 608002, India

**Keywords:** Air Pollution, Particulate Matter (PM2.5), Artificial Neural Network, Random Forest, Long Short-Term Memory

## Abstract

Introduction

Pollution of air in urban cities across the world has been steadily increasing in recent years. An increasing trend in particulate matter, PM _2.5_, is a threat because it can lead to uncontrollable consequences like worsening of asthma and cardiovascular disease. The metric used to measure air quality is the air pollutant index (API). In Malaysia, machine learning (ML) techniques for PM _2.5 _have received less attention as the concentration is on predicting other air pollutants. To fill the research gap, this study focuses on correctly predicting PM _2.5_ concentrations in the smart cities of Malaysia by comparing supervised ML techniques, which helps to mitigate its adverse effects.

Methods

In this paper, ML models for forecasting PM _2.5_ concentrations were investigated on Malaysian air quality data sets from 2017 to 2018. The dataset was preprocessed by data cleaning and a normalization process. Next, it was reduced into an informative dataset with location and time factors in the feature extraction process. The dataset was fed into three supervised ML classifiers, which include random forest (RF), artificial neural network (ANN) and long short-term memory (LSTM). Finally, their output was evaluated using the confusion matrix and compared to identify the best model for the accurate prediction of PM _2.5_.

Results

Overall, the experimental result shows an accuracy of 97.7% was obtained by the RF model in comparison with the accuracy of ANN (61.14%) and LSTM (61.77%) in predicting PM _2.5_.

Discussion

RF performed well when compared with ANN and LSTM for the given data with minimum features. RF was able to reach good accuracy as the model learns from the random samples by using decision tree with the maximum vote on the predictions.

## Introduction

Air pollution has become a major issue around the world, particularly in smart cities. Pollution of air is defined as any substance from any atmospheric source that continues to exist in any state and causes damage or the ability to alter the atmosphere’s typical characteristics, putting living things’ health at risk, or causing ecological disruption. ^1^

The concentration of air pollutants, particularly PM _2.5_, a very small particle with a diameter of 2.5 micrometers in the air, during the haze season in Malaysia determines the API readings [ http://apims.doe.gov.my/public_v2/aboutapi.html]. PM _2.5_, most dangerous among air pollutants, is a microscopic particle that can enter into lungs and have a negative consequence on the human respiratory system [ https://www.epa.gov/pm-pollution/particulate-matter-pm-basics].

Traffic pollution and industrialization are the primary sources of PM _2.5_ emissions. This stage of the PM _2.5_ problem can be seen in smart cities. In recent times, the size of population in the urban area has significantly risen because of industrialization and rural to urban area migration. The increase in population has increased in the modes of transportation and consumption of energy, which contributes to the growth of vehicles and industry in the urban city. ^2^

Several scientific investigations have shown that air quality is a major concern for smart cities and ML has started to emerge as a truly outstanding solution for predicting PM _2.5_ that takes the required steps to mitigate its negative effects, as the prediction must be accurate. However, in comparison with other countries, Malaysian PM _2.5_ prediction in the context of ML is not well established.

There has been significant progress in the prediction of PM _2.5_ concentrations in the air in smart cities around the world over the last decade, thus, using ML techniques to predict air PM _2.5_ concentrations in Malaysian smart cities could be beneficial. The goal of this research is to use ML to predict PM _2.5_ concentrations in the Air Pollutant Index (API) of six Malaysian smart cities. The sections that follow will be discussed and organized in the following order: Related works: this topic’s related works were discussed; Methods: to discuss the research methodology used to conduct this study; and Results and discussion: to discuss the study’s findings.

## Related works

Most cities in the world witnessed the levels of pollution have surpassed all the global standard guidelines in the past few decades of global modernization, resulting in existing issues. Because of the potentially fatal effects of PM _2.5_, research teams are working on developing a dependable method for analyzing PM _2.5_ concentrations in the polluted air. First, PM _2.5_ prediction approaches will be discussed, followed by a summary of related works in the second section.

### Approaches of PM _2.5_ prediction in smart cities

A research paper on air pollution forecasting using an ML model in Delhi’s smart cities was conducted. ^3^ ANN and support vector machine (SVM) algorithms were used as ML methods. Among the six SVM functions used to predict accuracy, medium Gaussian SVM had the highest accuracy.

Another study ^4^ predicted roadside PM _10_ and PM _2.5_ concentrations using ANN, Boosted Regression Trees (BRT), and SVM. The ML models were applied to pollutant, meteorological, and traffic data collected at nineteen London Air Quality Monitoring (AQM) sites between 2007 and 2012. The ANN and BRT models performed well. Shahriar et al. ^5^ evaluated the effectiveness of Autoregressive Integrated Moving Average (ARIMA)-SVM and ARIMA-ANN, for daily PM _2.5_ prediction in Bangladesh. The ARIMA-ANN and CatBoost models performed well when the hybrid models were compared with DT, Catboost.

The daily PM _2.5_ levels in the Greater London area was predicted using an ensemble ML approach. ^6^ Random forest (RF) was one of the ML models of the ensemble. RF outperformed the k-nearest neighbor (KNN) and gradient boosting machine (GBM). With a ten-fold cross-validated R _2_ of 0.828, the model performed exceptionally well. A comparison study of ML methods for predicting PM _2.5_ concentrations in smart cities was successfully undertaken in Malaysia. ^7^ Multi-layer perceptron (MLP) and RF were the techniques used. RF was effective, according to the findings of this study.

Deep learning (DL) models, which are algorithms inspired by the function and structure of the brain, have also been studied for PM _2.5_ predictions. In the study, ^8^ hybrid model long short-term memory (LSTM)-convolutional neural network (CNN) hybrid model performed well compared to the hybrid gated recurrent Unit (GRU)-CNN. In another research, ^9^ the proposed deep neural network (DNN)-LSTM hybrid model, was effective when compared with multiple additive regression trees (MART) and deep feedforward neural network (DFNN). Zhang et al. ^10^ suggested a DL model, which included bidirectional LSTM (Bi-LSTM) and an auto-encoder (AE) for the PM _2.5_ prediction.

Following the review of papers, it is evident that supervised ML models are considered for PM _2.5_ predictions. Next, ANN, LSTM and RF are the repeatedly used models for the prediction of PM _2.5_ but were not compared. Nonetheless, in comparison to the rest of the world, Malaysia has limited studies. As a result, the authors proposed using ML to predict air quality (PM _2.5_).

## Methods

The methods for determining the research’s outcome are discussed in this section.

### Problem definition

In the world of smart cities, haggling with pollution of air is one of the most pressing issues in the environment. In Malaysia, smart cities such as Kuala Lumpur, Johor Bharu, Penang, Putrajaya, Kota Kinabalu, and Kuching have experienced severe pollution of air. Air pollution forecasting has become a significant strategy for Malaysia’s Department of Environment in preventing air pollution. As a result, ML must be utilized to determine the PM _2.5_ prediction’s accuracy.

### Data collection

The data was obtained from the Malaysia’s Department of Environment. ^2^ The data was collected between 2017 and 2018. PM _2.5_ readings have been taken since July 2017, according to the Department of Environment. The data were gathered from the air quality monitoring stations, including: Batu Muda and Cheras (Wilayah Persekutuan Kuala Lumpur); Batu Pahat, Kota Tinggi, Kluang, Larkin, Muar, Pasir Gudang, Pengerang, and Segamat (Johar Bharu); Seberang Jaya, Seberang Prai, Minden, and Balik Pulau (Penang); Putrajaya; Kota Kinabalu (Sabah); and Kuching (Sarawak).

The data span the months of July 2017 to December 2018. Beginning in July 2017, the DOE began taking readings of PM _2.5_ concentrations in the air. The features included in the dataset were station id, location, ate and PM _2.5_.

### Data pre-processing

DOE provided clean datasets with no noisy data, such as missing data or outliers. As a result, data transformation was carried out. The data types were transformed to integers during data transformation so that the classification algorithm could operate on them. To convert the categorical variables station id and location to integers, they were encoded.

In addition, the data is in the form of time series. The date was thus divided into the following sections: day, day of the year, week of the year, month, quarter, and weekdays. In order to improve ML accuracy, this procedure is taken to measure various points.

Following a review of the PM _2.5_ values, it was decided to introduce a new column called Label. The air quality status indication is defined by the label column is relies on the Malaysia’s Air Quality Status Indication rating. The target column for ML to forecast values will be the label column.

The data factors were converted to numerical data. The data is formatted as integer as it has to be fed into the supervised training classifiers.

### Feature selection

A key stage that must be accomplished is the identification of relevant characteristics that are connected to the target variable. The accuracy of the classifier will increase as a result of picking the best feature. On this dataset, the SelectKBest technique was employed. The characteristics are picked based on the k number, which decides which features receive the greatest ratings. In this work, chi-squared statistics for classification tasks are evaluated using the chi2 function.

Following the implementation of the approaches, five characteristics are chosen: PM _2.5_, Station Id, location, day of the year, and day of the year. These five characteristics, according to the chi-square scores, show a significant relationship with the target feature, which is the "Label." These characteristics will be utilised to train the model, resulting in greater accuracy when assessing the real PM _2.5_ label values.

### Test-train data and classification

For this experiment, the train-test split is 60-40. The data with labels is used to train and test the classifiers. Supervised ML algorithms ANN, LSTM and RF were used to predict PM _2.5_ accurately.

First, Artificial Neural Network (ANN): The interconnection of assembly of nodes with their structure using a directed link [ https://www.mathworks.com/discovery/neural-network.html]. It has three layers: an input layer, a concealed layer, and an output layer. To begin, 10 neurons will be in the input layer, 200 neurons in the hidden layer and their output layer will have ten neurons. To determine the number of epochs to update the network weights during training, the maximum number of iterations was set to 100. Adam, which works well on large datasets, was the solver employed. Finally, for this dataset, inactivation parameters, ReLU, were used. ReLU allows the model to run faster, especially in backpropagation. After the model has been set up, the predicted values are obtained by running it against test data.

Next, LSTM: recurrent neural networks (RNNs) have a long-term dependency problem that LSTM networks were created to solve due to the vanishing gradient problem. LSTMs differ from more traditional feedforward neural networks by having feedback connections. This trait allows LSTMs to process whole data sequences without having to treat each point in the sequence separately, instead storing important information from prior data in the sequence to aid in the processing of new data points [ https://towardsdatascience.com/lstm-networks-a-detailed-explanation-8fae6aefc7f9].

This model’s LSTM has three layers, each of which takes the output of the previous LSTM and outputs a prediction for the next time step. The three *LSTMCell* objects that were created were encapsulated using *MultiRNNCell* in TensorFlow. The model is run against test data after it has been set up.

Finally, random forest: a model comprised of hundreds or thousands of decision trees [ https://builtin.com/data-science/random-forest-algorithm#how]. Each one is trained with a random dataset and splits the nodes in each tree by few features. The n estimator parameter is set to 100, implying that 100 decision trees will be constructed. The model trains each tree before determining the best predicted values, which are determined by the number of votes. Next, to acquire the expected values, the model is run on the test data.

### Confusion matrix

The performance of a ML model is assessed with the confusion matrix. The confusion matrix will display the comparison between the actual and predicted values of the ML models [ https://www.analyticsvidhya.com/blog/2020/04/confusion-matrix-machine-learning/]. The evaluation’s goal is to see how well our model performed in terms of ML. The result of the ANN, LSTM and RF are analyzed with confusion matrix. There are four parts to the confusion matrix:

1. True positive (TP) indicates that the actual and predicted values are same. Both are positive values.
2. True negative (TN): The real values are predictable. Even if the real value is positive, negative is predicted. The expected value is improperly forecasted.
3. False positive (FP): Although the actual value is negative, the predicted value is positive. The expected value is improperly forecasted.
4. False negative (FN): Incorrect forecasting of a value. The real value is positive, however, but is expected to be negative.

These equations are derived from the confusion matrix in order to determine ML performance.

$$\text{Accuracy}=\frac{\mathrm{TP}+\mathrm{TN}}{\mathrm{TP}+\mathrm{FP}+\mathrm{TN}+\mathrm{FN}}$$ (1)

$$\mathrm{Precision}=\;\frac{\mathrm{TP}}{\mathrm{TP}+\mathrm{FP}}$$ (2)

$$\text{Recall}=\frac{\mathrm{TP}}{\mathrm{TP}+\mathrm{FN}}$$ (3)

Random forest, LSTM, and ANN are compared in this study because these ML algorithms have shown to be more accurate in many studies, as discussed in the related work. Since limited research on pollution of air (PM _2.5_) in Malaysia has been done, in this study, ML methods will be analysed, and the best ML method will be chosen. This study will be conducted entirely in Python. Anaconda Jupyter Notebook is used to stimulate the module.

## Results and discussion

The results of each classifier will be discussed and justified in this section.

### ANN *versus* LSTM *versus* random forest

**Figure 1.**
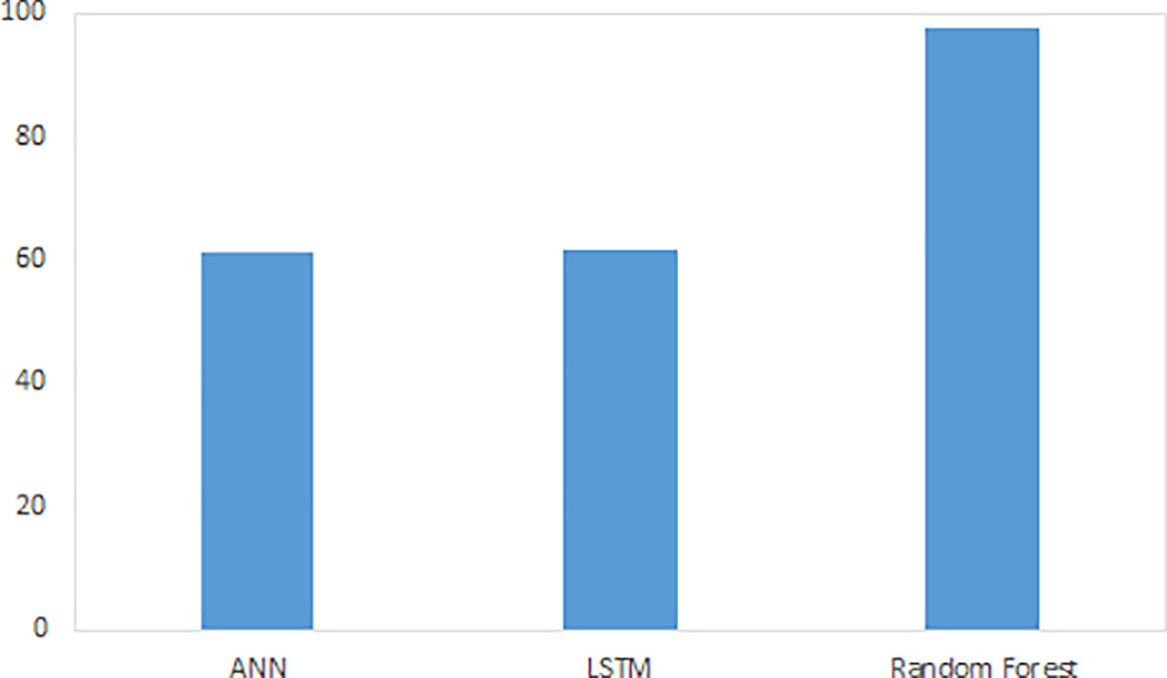
Accuracy of ML models.

The ML algorithms were employed on the DOE data, which has few features for the data over the period 2017 to 2018. The ANN and LSTM predict PM _2.5_ with an accuracy of 61.14% and 61.77% respectively and cannot improve further after 150 epochs as they require more data to get a significant conclusion. Though ANN, LSTM and RF have 100 layers, the accuracy of RF (97.7%) for PM _2.5_ predictions was higher as the model learns from the random samples by using decision tree with the maximum vote on the predictions, which improves the interpretation unlike ANN and LSTM, that decimate the interpretability of the features. Figure 1, shows the accuracy of the comparison algorithms. Overall, the research results reveal that the RF model outperformed ANN and LSTM.

## Conclusion and future work

In this paper, air pollution prediction using ML models were tested in Smart Cities of Malaysia because of the severity of air pollution in urban areas. Only supervised ML techniques were used for comparison in this paper to predict PM _2.5_. Previous research has used supervised ML techniques to predict PM _2.5_ levels. However, comparative analysis of them is often required for the identification of the best model for accurately forecasting the concentration of PM _2.5_. When compared, RF outperformed ANN and LSTM in predicting PM _2.5_ in Malaysian Smart Cities with an accuracy of 97.7% for a dataset with less data and features.

## Data availability

DANS: Underlying data for ‘Machine learning methods to predict particulate matter PM _2.5_’. https://doi.org/10.17026/dans-2zd-rgue

Data are available under the terms of the Creative Commons Attribution 4.0 International license (CC-BY 4.0).

## Author contributions

Rishanti and Kuhaneswaran did the conception of the work, data collection, data analysis and interpretation, drafting the article, and revision to the final version under the guidance of S Subramanian and their supervisors Su-Cheng Haw and Palanichamy Naveen. Palanichamy Naveen is the corresponding author for this paper.
