# Supplementary material for: Machine learning methods to predict particulate matter PM 2.5
Source: F1000Res. 2022 Apr 11;11:406. [Version 1] doi: 10.12688/f1000research.73166.1 (PMC9723408; doi:10.12688/f1000research.73166.1)
Supplement: Peer review history

# Peer review: Reviewer response for version 1

**DOI:** 10.5256/f1000research.76798.r156478

**Published:** 2022-12-05

**Authors:** Charles Raja S.

**Affiliations:** 1 Department of Electrical and Electronics Engineering, Thiagarajar College of Engineering, Madurai, India

The paper discussed the topic of Machine learning methods to predict particulate matter PM2.5.

 The authors have done an extensive literature survey and they have collected real-time data to train the ML program.

 The authors are requested to clarify some minor clarifications:

 •    Kindly explain how you performed pre-processing for your real-time data set.

 •    What are the features available in the considered data set?

 •    Mention the reason for labelling the data.

 •    Write the accuracy of the methods in terms of RMSE values if possible.

Is the work clearly and accurately presented and does it cite the current literature?

Yes

If applicable, is the statistical analysis and its interpretation appropriate?

Yes

Are all the source data underlying the results available to ensure full reproducibility?

Yes

Is the study design appropriate and is the work technically sound?

Yes

Are the conclusions drawn adequately supported by the results?

Yes

Are sufficient details of methods and analysis provided to allow replication by others?

Yes

Reviewer Expertise:

Machine learning, Deep learning and Smart grids.

I confirm that I have read this submission and believe that I have an appropriate level of expertise to confirm that it is of an acceptable scientific standard.

# Peer review: Reviewer response for version 1

**DOI:** 10.5256/f1000research.76798.r134550

**Published:** 2022-05-12

**Authors:** Stella Morris

**Affiliations:** 1 Department of Electrical Engineering, Universiti Tunku Abdul Rahman, Petaling Jaya, Malaysia

This article focuses on the prediction of PM _2.5_ concentrations in Malaysia which would help to combat air pollution.

 An increase in particulate matter, or PM _2.5_, is a hazard since it can have unpredictable implications such as asthma and cardiovascular disease worsening. Machine learning (ML) techniques have gotten less attention in Malaysia because the focus is on forecasting other air contaminants. Smart cities are concerned about air pollution. PM _2.5_ emissions are mostly caused by traffic pollution and industrialization.

 This study has applied machine learning to estimate the Air Pollution Index (API) of six Malaysian smart cities, among other things. An ensemble ML technique was used to forecast daily PM _2.5_ levels in the Greater London area. Random forest (RF) was one of the ensemble's machine learning models. The RF machine outperforms the KNN and gradient boosting machines (GBM).

 Smart cities in Malaysia have suffered from significant air pollution. Therefore, this proposed machine learning approach would help to improve the accuracy of the PM _2.5_ prediction and hence mitigate air pollution.

 The investigation has been conducted using data sets from 2017 to 2018. To validate their effectiveness, those predicted outputs could be compared to the most recent available data sets.

Is the work clearly and accurately presented and does it cite the current literature?

No

If applicable, is the statistical analysis and its interpretation appropriate?

I cannot comment. A qualified statistician is required.

Are all the source data underlying the results available to ensure full reproducibility?

Yes

Is the study design appropriate and is the work technically sound?

Yes

Are the conclusions drawn adequately supported by the results?

Yes

Are sufficient details of methods and analysis provided to allow replication by others?

Yes

Reviewer Expertise:

Power system control, stability, and optimization

I confirm that I have read this submission and believe that I have an appropriate level of expertise to confirm that it is of an acceptable scientific standard.
